# Performance optimization of efficient PbS quantum dots solar cells through numerical simulation

**DOI:** 10.1038/s41598-023-36769-y

**Published:** 2023-06-29

**Authors:** Sandeep Kumar, Pragya Bharti, Basudev Pradhan

**Affiliations:** 1 Department of Energy Engineering, Central University of Jharkhand, Brambe, Ranchi, 835205 Jharkhand India; 2 Centre of Excellence (CoE) in Green and Efficient Energy Technology (GEET), Central University of Jharkhand, Brambe, Ranchi, 835205 Jharkhand India

**Keywords:** Energy science and technology, Materials science

## Abstract

Colloidal quantum dots (CQDs) solar cells are less efficient because of the carrier recombination within the material. The electron and hole transport layers have high impact on the performance of CQDs based solar cells which makes its investigation a very important component of the development of the more efficient devices. In this work, we have tried performance optimization in tetrabutyl ammonium iodide capped lead sulfide (PbS) CQDs (PbS-TBAI) as absorber layers based solar cells by incorporating different hole transport layers (HTLs) to achieve better power conversion efficiency (PCE) in different device architectures by SCAPS—1D numerical simulation software. It was observed from the simulation that the ITO/TiO_2_/PbS-TBAI/HTL/Au device architecture shows higher power conversion efficiency as compared to the conventional experimentally realized device architecture of ITO/TiO_2_/PbS-TBAI/PbS-EDT/HTL/Au. The influence of interface defect density (IDD) at the interface TiO_2_/PbS-TBAI has also been studied where IDD is varied from 1 × 10^13^ cm^−2^ to 1 × 10^18^ cm^−2^ while keeping the rest of the device parameters intact. The result shows a noteworthy reduction in the PV performance of the device at higher IDD. This modelled device structure provides a new direction toward the experimental realization in high efficiency PbS QDs solar cells.

## Introduction

In order to meet the escalating demand of a cost effective, durable and highly efficient solar cell technology, it is of paramount importance to find a device architecture that can meet the above demands. Crystalline Si based solar cell technology has reached its threshold in the last decade reaching a maximum of 26.8% which is close to the theoretical efficiency limit^1^. Though c-Si has mature synthesis process but its exorbitant price has always been a matter of concern. Several researchers are working to equipoise cost and efficiency aspect of solar cell by incorporating various low cost semiconducting materials in a device architecture that can be used as an efficient solar cell technology. Colloidal quantum dots (CQDs) due to their property of tunable bandgap, multiple exciton generation (MEG), near infrared (NIR) absorption etc. have emerged as a vital material for solar cells applications. This property of Quantum dots (QDs) has a great advantage on the design of the solar cell, since optoelectronic performance can be tuned by changing the bandgap, and thereby can utilize the solar spectrum more efficiently which makes it a potential candidate to be used in tandem solar cell devices^2,3^. Among various CQD materials, lead sulfide (PbS) CQDs have proved to be the most prominent material for QDs based solar cell with highest performance^4–6^. PbS CQDs are group IV and VI compound semiconductor that have been of keen interest of various researchers due to their promising properties of multiple exciton generation^7,8^, large bandgap tunability^9^ and NIR absorption^10^. These properties have made PbS CQDs a propitious material for various application like photo-detectors^11^, cell imaging^12^, light emitting diodes^13^ and solar cells^14,15^. For improvement of solar cell performance, research has been emphasized on synthesis of different bandgap colloidal quantum dots varying size^16^ which can easily be tuned for optimal light absorption^17^. The CQDs based solar cells are one of the promising candidates among available 3rd generation solar cell market^18,19^. So far the highest achieved 13.8% PCE in PbS CQDs based solar cell experimentally using monolayer perovskite bridges between CQDs^20^. Still this device suffers from low carrier transport and further improvement is necessary for its large scale commercialization.

CQDs can be synthesized using solution processing which enables the realization of high efficiency solar cells^21^. The important concern is that the performance of these QDs based solar cells is not up to the mark compared to the other PV technologies due to carrier recombination within the quasi-neutral region (QNR) near the electron (hole) collecting interfaces^22^. In general, PbS QDs based solar cell is based on depleted hetero-junction design and consists of light-absorbing material such as PbS CQDs, thiol-treated CQDs sandwich between n-type metal oxide as electron transport layer (ETL) and hole transport layer (HTL). Although, the internal electric field around metal oxide/PbS QDs interface extracts the majority of light generated carriers, the hole transport layer also plays important role as better transport of holes improves generation of current hence device efficiency is improved. Along with experiments, different simulation based on various photophysical properties of the constituent materials, also plays a critical role in analyzing device performance without wasting lots of materials as well as time. In this study we designed and simulated using tetrabutylammonium iodide (TBAI) capped colloidal PbS CQDs layer as absorber layer and TiO_2_ as ETL and various hole transport materials for further enhancement of device performance through SCAPS—1D numerical simulation software. Different device architectures of PbS CQDs based solar cells are also explored. We have compared their relative efficiencies keeping rest of the device structure and properties constant and have found best alternative which can help researchers to fabricate high efficiency PbS CQDs based solar cell in real experimental conditions.

### Device structure and method

**Figure 1:**
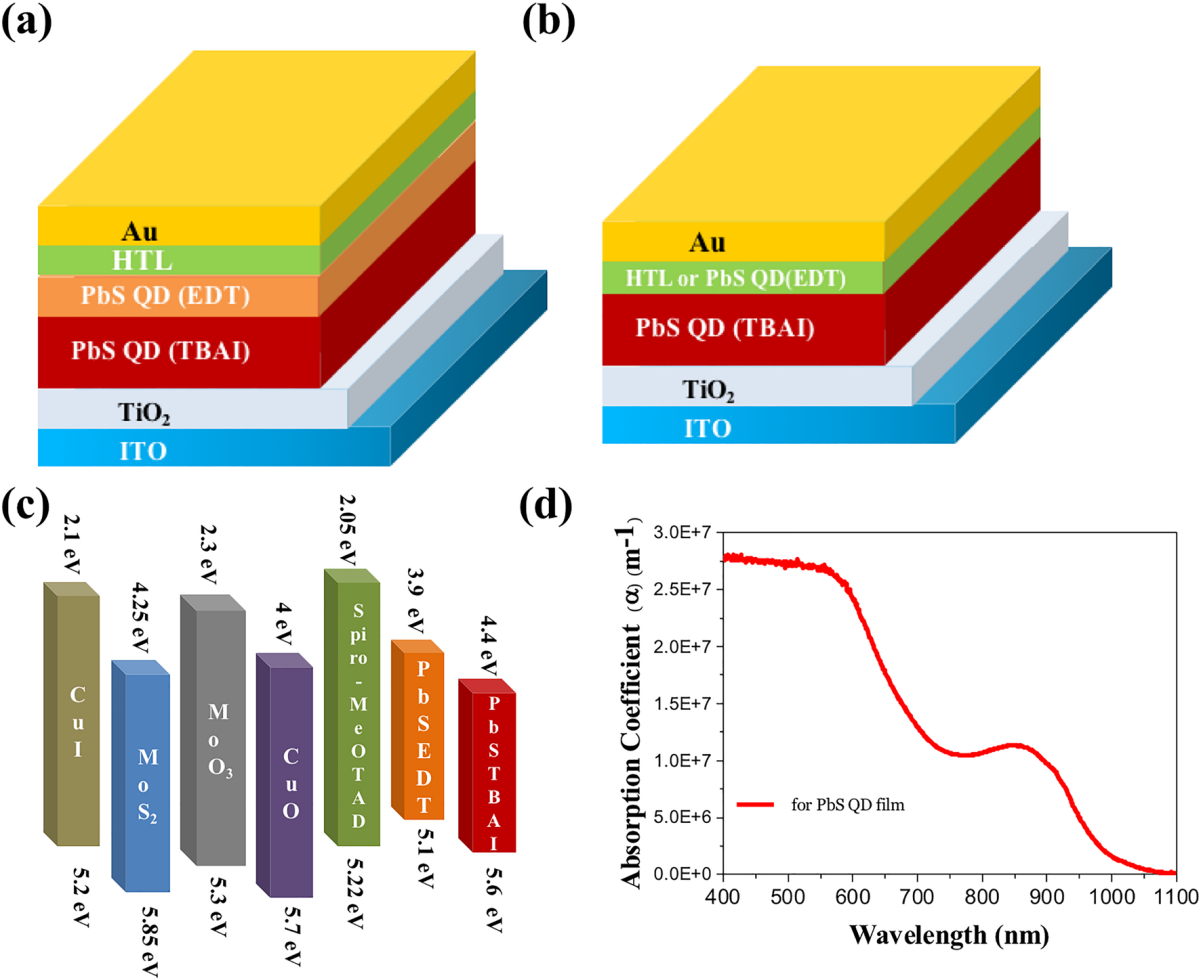
Schematic structure of (**a**) architecture—1(ITO/TiO_2_/PbS-TBAI/PbS-EDT/HTL/Au) (**b**) architecture—2 (ITO/TiO_2_/PbS-TBAI/HTL/Au). (**c**) Energy band diagram of PbS QDs solar cell with different HTLs. (**d**) Wavelength dependent absorption coefficient of PbS QD thin film.

So far experimentally successful conventional device architecture of ITO/TiO_2_/PbS-TBAI/PbS-EDT/HTL/Au (architecture—1) as shown in the Fig. 1a, where PbS TBAI is used for the main absorber layer, Titanium dioxide (TiO_2_) as ETL, and addition layer PbS-EDT for hole extraction which allows holes to pass through it easily due to favorable band alignment with the active PbS-TBAI layer. For this simulation work, the optimum layer thickness of PbS-TBAI and PbS-EDT are taken from the research works^23,24^. For the ETL, we have fixed TiO_2_, as it has shown improvement in the efficiency of the PbS CQDs based solar cell reaching an efficiency of about 13.94%^22^. These two-material layers of ETL and HTL are used to extract the photo-generated electron and hole to the respective electrodes. After calibration for the conventional device architecture, different HTLs, are introduced for analysis. In another device architecture of ITO/TiO_2_/PbS-TBAI/HTL/Au (architecture—2), we have removed the PbS—EDT layer from the conventional architecture and replaced with different HTLs like Copper(I) iodide (CuI), Molybdenum disulfide (MoS_2_), Molybdenum oxide (MoO_3_), 2,2′,7,7′-Tetrakis[*N*,*N*-di(4-methoxyphenyl)amino]-9,9′-spirobifluorene (Spiro-MeOTAD), and Copper(II) oxide (CuO) after the active PbS-TBAI layer, as shown in Fig. 1b for further optimization of the device performances. The light incidents through the ITO end and acts as front contact and gold (Au) as back metal contact. The corresponding energy band diagram with different HTLs is illustrated in the Fig. 1c which helps to understand the flow of electron and holes through different layers of the device. The detailed comparative analysis has been made between both the architectures using numerical simulation under AM 1.5G 1 sun spectra. We have done one-dimensional simulation analysis using SCAPS-1D (Solar Cell Capacitance Simulator) tool, developed by Department of Electronics and Information Systems (ELIS) of the University of Gent, Belgium^25^. The SCAPS-1D is very useful tool to simulate device performance before doing real experiment using different parameters of constituent layers^26^. With the help of this SCAPS simulation tool, we can calculate current/voltage characteristics, photovoltaic parameters, quantum efficiencies, carrier density profile, total generation/recombination profile, corresponding energy band diagrams, etc. It works based on the one dimensional Poisson’s equation in semiconductors, carrier continuity (electron/hole transport), and the drift–diffusion differential equations. The Poisson’s equation is a relationship of the electric field (*E*) as follows in Eq. (1)

1 $$\frac{{d^{2} \psi }}{{dx^{2} }} = \frac{\partial E}{{\partial x}} = - \frac{\rho }{\varepsilon } = \frac{q}{\varepsilon }\left[ {p - n + N_{D}^{ + } - N_{A}^{ - } } \right]$$

where $\psi$ is electrostatic potential, *p*, *n* are holes and electrons concentration respectively, *q* is the elementary charge, $N_{D}^{ + }$ and $N_{A}^{ - }$ are ionized donor and acceptor dopant carrier concentrations respectively, *ε* is dielectric constant. The continuity equation for electron and hole are as follows in Eqs. (2) and (3)

2 $$\frac{{\partial J_{n} }}{\partial x} + G_{n} - U_{n} \left( {n,p} \right) = 0$$

3 $$- \frac{{\partial J_{p} }}{\partial x} + G_{p} - U_{p} \left( {n,p} \right) = 0$$

Charge carriers drift–diffusion equations are indicated by Eqs. (4) and (5).

4 $$J_{n} = qn\mu_{n} E + qD_{n} \frac{\partial n}{{\partial x}}$$

5 $$J_{p} = qp\mu_{p} E - qD_{p} \frac{\partial p}{{\partial x}}$$

where *D*_*n*_ and *D*_*p*_ are electron and hole diffusion coefficient, *J*_*n*_ and *J*_*p*_ are current densities of electron and holes, *G* is generation rate (electron and holes) and *U*_*n*,*p*_ is the net recombination rate, *μ*_*p*_, *μ*_*n*_ are carrier mobility for hole and electron respectively and E is the electric field. The relationship between diffusion coefficient and carrier mobility is represented by Einstein relation as given in Eq. (6).

6 $$D\left( {n,p} \right) = \frac{{k_{B} T}}{q}\mu \left( {n,p} \right)$$

where, $D(n,p)$ is diffusion coefficient (m^2^s^−1^), $\mu$ is electrical mobility (m^2^V^−1^s^−1^), k_B_ is a Boltzmann constant, *q* is electric charge, and T is absolute temperature (K).

Major attention is given to optimize different parameters in a way through which we can get a clear insight of device performances. The Table 1 shows the different layer parameters that have been used for this simulation process. Some values have been derived from the already published papers while the others have been optimized within the feasible limit after studying the impacts of different structural properties on the device performance.

**Table 1:** Input parameters of different materials for PbS QD based solar cells^22^ used in simulation.

Parameter	PbS-TBAI	PbS-EDT	TiO_2_	CuI	MoS_2_	MoO_3_	CuO	Spiro-MeOTAD
Thickness (µm)	220/265	45	80	10	10	10	10	10
Bandgap (eV)	1.14	1.14	3.2	2.98	1.29	3	1.5	3.17
Electron affinity (eV)	4	3.9	3.9	2.1	4.2	2.5	4.07	2.05
Relative dielectric permittivity	20	20	9	6.5	3	12.5	18.1	3
CB effective density of states (cm^−3^) × 10^18^	10	10	2.2	10^4^	2.2	2.2	22	10^2^
VB effective density of states (cm^−3^) × 10^19^	1	1	1.8	10^3^	1.8	1.8	55	10
Electron thermal velocity (cm s^−1^)	10^7^	10^7^	10^7^	10^7^	10^7^	10^7^	10^7^	10^7^
Hole thermal velocity (cm s^−1^)	10^7^	10^7^	10^7^	10^7^	10^7^	10^7^	4.16 × 10^7^	4.16 × 10^7^
Electron mobility (cm^2^V^−1^s^−1^)	0.02	2 × 10^–4^	20	1.69 × 10^–4^)	100	25	100	2
Hole mobility (cm^2^ V^−1^s^−1^)	0.02	20	10	1.69 × 10^–4^	150	100	0.1	0.01
N_D_ grading (uniform Acceptor density (cm^−3^)	10^15^	10^14^	10^17^	10^14^	10^14^	0	0	0
N_A_ grading (uniform donor density (cm^−3^)	10^15^	10^16^	0	10^14^–10^19^	10^14^–10^19^	10^14^–10^19^	10^14^–10^19^	10^14^–10^19^
Total defect density (N_t_)	10^14^	10^14^	10^14^	10^14^	10^14^	10^14^	10^14^	10^14^

For realistic simulation and analysis of semiconductor devices, we need to incorporate accurate and reliable optoelectronic properties for all the materials used in the simulation. We have used neutral defect type with 10^–19^ cm^−2^ electron as well as hole capture cross section, reference defect level above the highest *E*_*v*_ having energy of 0.6 eV, and total defect density of 2 × 10^14^ cm^−3^ at PbS QD/TiO_2_ interface for consideration of interfacial defect density(IDD). Also the role of mobile ions cannot be considered in analysis because of software limitations^27^. The absorption coefficient of PbS used in the simulation as shown in Fig. 1d is obtained from our experimental measurement^28^.

## Results and discussion

### Influence of doping density

**Figure 2:**
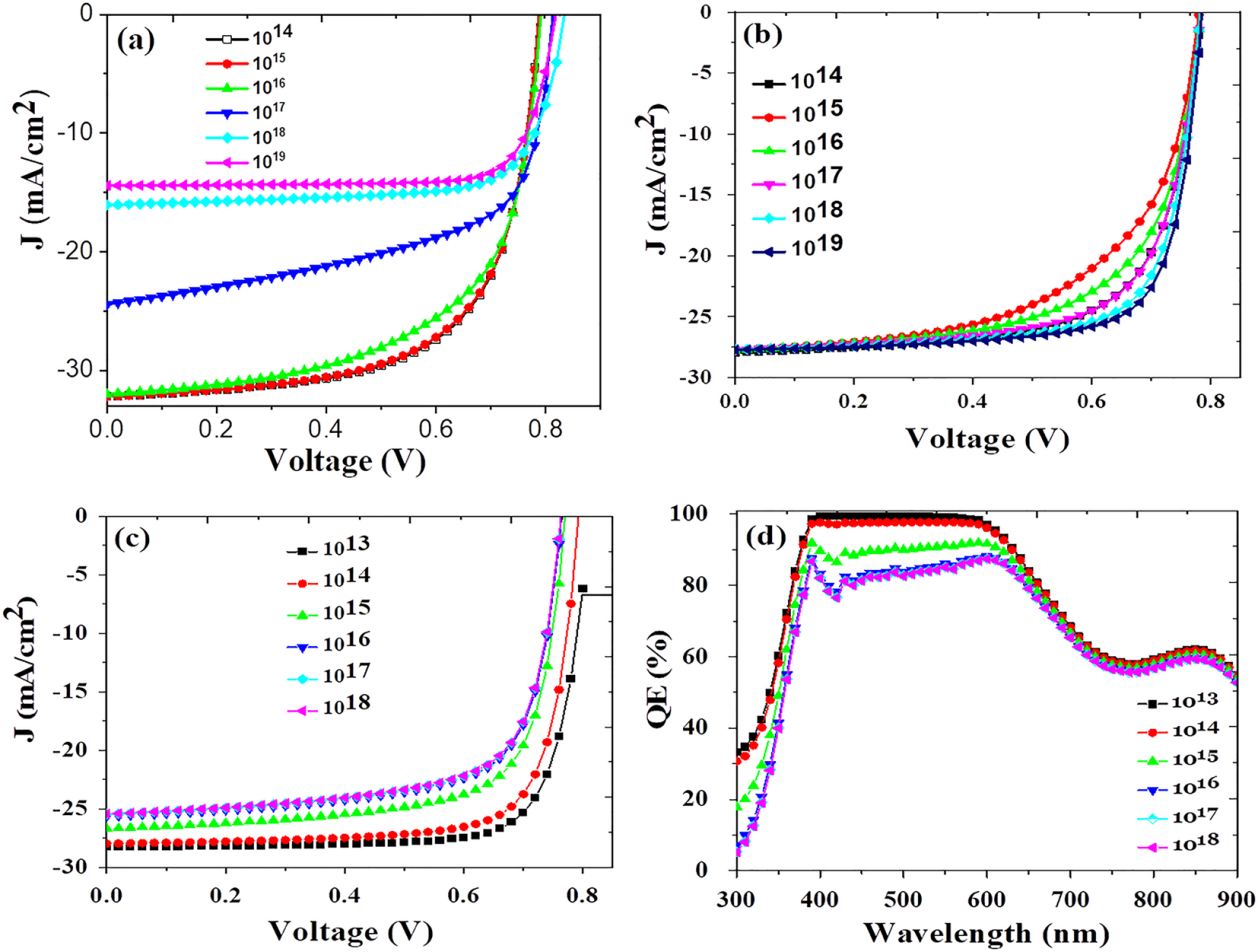
*J–V* characteristics of the device simulated with conventional architecture ITO/TiO_2_/PbS-TBAI/PbS-EDT/MoO_3_/Au, (**a**) with variation in donor doping density from 10^14^ to 10^19^ cm^−3^ of the absorber layer (PbS-TBAI). (**b**) With variation in acceptor doping density from 10^14^ to 10^19^ cm^−3^ of the p-type layer (PbS-EDT). (**c**) J–V characteristics of the device with variation in IDD from 10^13^ to 10^18^ cm^−3^. (**d**) QE spectra of devices with variation in IDD from 10^13^ to 10^18^ cm^−3^ at PbS-TBAI/TiO_2_ interface.

**Figure 3:**
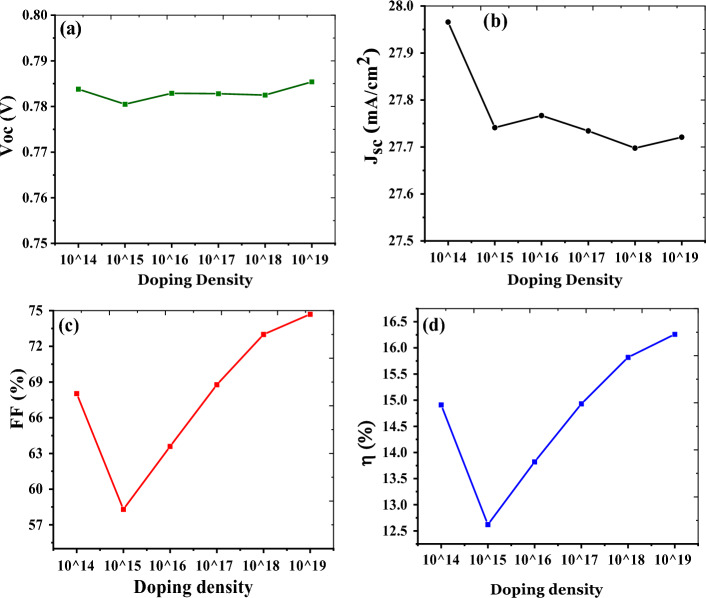
Variation in device parameters with acceptor doping density with fixed IDD of 2 × 10^14^ cm^−3^ (**a**) V_OC_ vs acceptor doping density, (**b**) J_sc_ vs acceptor doping density, (**c**) FF vs acceptor doping density, (**d**) efficiency vs acceptor doping density of the active layer.

The numerical simulations of PbS CQDs based solar cells of different architectures with different layers were performed based on tabulated parameters collected from different theoretical and experimental research papers. First we have studied the impact of donor doping density of the active layer (PbS-TBAI) on device performances in conventional architecture-1, ITO/TiO_2_/PbS-TBAI/PbS-EDT/MoO_3_/Au. Figure 2a shows the current–voltage characteristics of device with different donor doping density which is varied from 10^14^ to 10^19^ cm^−3^ keeping all the other parameters same. It was observed that as the doping increases in the active layer, the device short-circuit current decreases and in the same time open circuit voltage increase. Ultimately device efficiency decreases with the donor doping density. The reverse saturation current decreases as donor doping concentration increases in the active layer. On the other hand, as doping concentration increase the built in potential (*V*_bi_), also increase, because of this open circuit voltage (V_OC_) of the device also increase^29,30^. In the case of short circuit current densities (J_SC_), at lower doping concentration, photogenerated carrier collection is higher due the presence of wide depletion region. As doping concentration increases, depletion region width decreases, which reduces the carrier collection leading to lower Jsc. To obtain optimized device performance, doping concentration of the different transport layers play very important role especially in carrier transport. Here, in this work we have tried to understand the influence of acceptor doping concentration in conventional device architecture on all of the devices. The acceptor concentration has been varied from 1 × 10^14^ cm^−3^ to 1 × 10^19^ cm^−3^, while keeping other device parameters fixed, under which, the PV performances using J–V characteristics of all the devices is evaluated and represented in Fig. 2b. It is observed that with the increase in the acceptor doping concentration of the PbS-EDT, the performance of the device has improved very significantly as shown in Fig. 3a–d. A small change in the J_SC_ of the device is observed (Fig. 3b); however, the V_OC_ remains almost constant about 0.77 V and fill factor (FF) increase significantly as the acceptor doping of the PbS-EDT is increased as shown in Fig. 3a and c respectively. FF is mainly affected by the series resistance of the device. The total series resistance of the device is combination of the resistance of individual layers and their associated interfaces and metal—semiconductor contacts contribute into it. As the PbS-EDT doping is increased, the resistivity of PbS-EDT decreases and hence supports the hole flow from the absorber layer to HTL easily. The higher built-in potential and electric field with doping of 1 × 10^19^ cm^−3^ are validated by a higher slope of quasi-Fermi energy levels, this higher slope results in improved V_OC_. The fill factor of the device increases with increase in acceptor doping density from 68.03% at 10^14^ cm^−3^ to 74.70% at 10^19^ cm^−3^, which ultimately helps holes to move easily to the contact electrode as shown in Fig. 3c. The efficiency of the solar cells is directly related to the FF, as the FF increase, the efficiency of the device increases and hence the cell achieved 16.26% efficiency at 10^19^ cm^−3^ doping density as shown in Fig. 3d.

**Figure 4:**
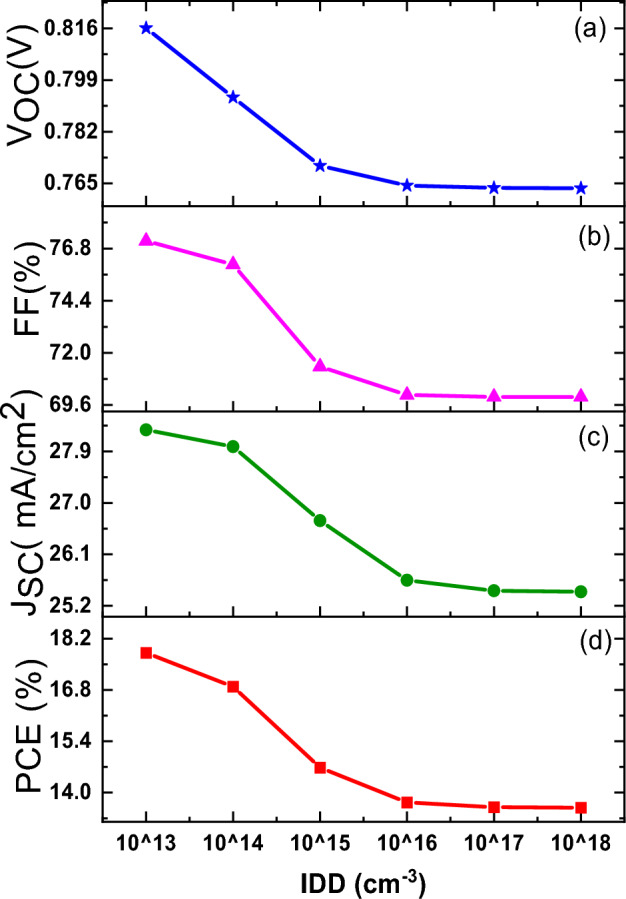
Variation in, (**a**) V_OC_, (**b**) *FF*, (**c**) J_sc_, and (**d**) power conversion efficiency (PCE) with varying IDD at TiO_2_/PbS-TBAI interface which clearly shows the decline in all performance related parameters with increase in IDD.

Further, the conventional device architecture has been simulated with varying the interface defect density at TiO_2_/PbS-TBAI interface. The effect of interface variation can be observed at light entering side of the device i.e. at TiO_2_/PbS-TBAI interface. The effect of variation in interface defect density (IDD) where light reaches later after the main absorber layer is negligible^22^. The defect density at TiO_2_/PbS-TBAI layer is varied from 10^13^ to 10^18^ cm^−3^ by keeping all other parameters fixed. Figure 2c and d show the J–V characteristics and correspoing QE spetra for different interface defect density. It was observed that there exists significant decrease in charge carrier extraction due to increase in defect density within 650 nm wavelength, beyond that the changes are insignificant. As the light enters the device through TiO_2_ layer side, most of the high energy photons get abosrbed at the TiO_2_/PbS-TBAI interface and create electron–hole pairs. But due to higher defect density, the recombination of generated charge carrier is also higher at the TiO_2_/PbS-TBAI interface. All the three parameters V_OC_, Jsc and FF reduces as the IDD increases with same nature which is shown in the Fig. 4a–d. This results in reduction of the device efficiency from 17.81% at IDD 10^13^ cm^−3^ to 13.59% at IDD 10^18^ cm^−3^ which clearly shows increased carrier losses due to increment in IDD.

Further we have examined the performance of devices having architecture-1 (ITO/TiO_2_ (80 nm)/PbS-TBAI (220 nm)/PbS-EDT (45 nm)/HTL (10 nm)/Au) by having different HTLs over the architecture-2 (ITO/TiO_2_(80 nm)/PbS-TBAI (220 nm)/HTL/Au).

**Figure 5:**
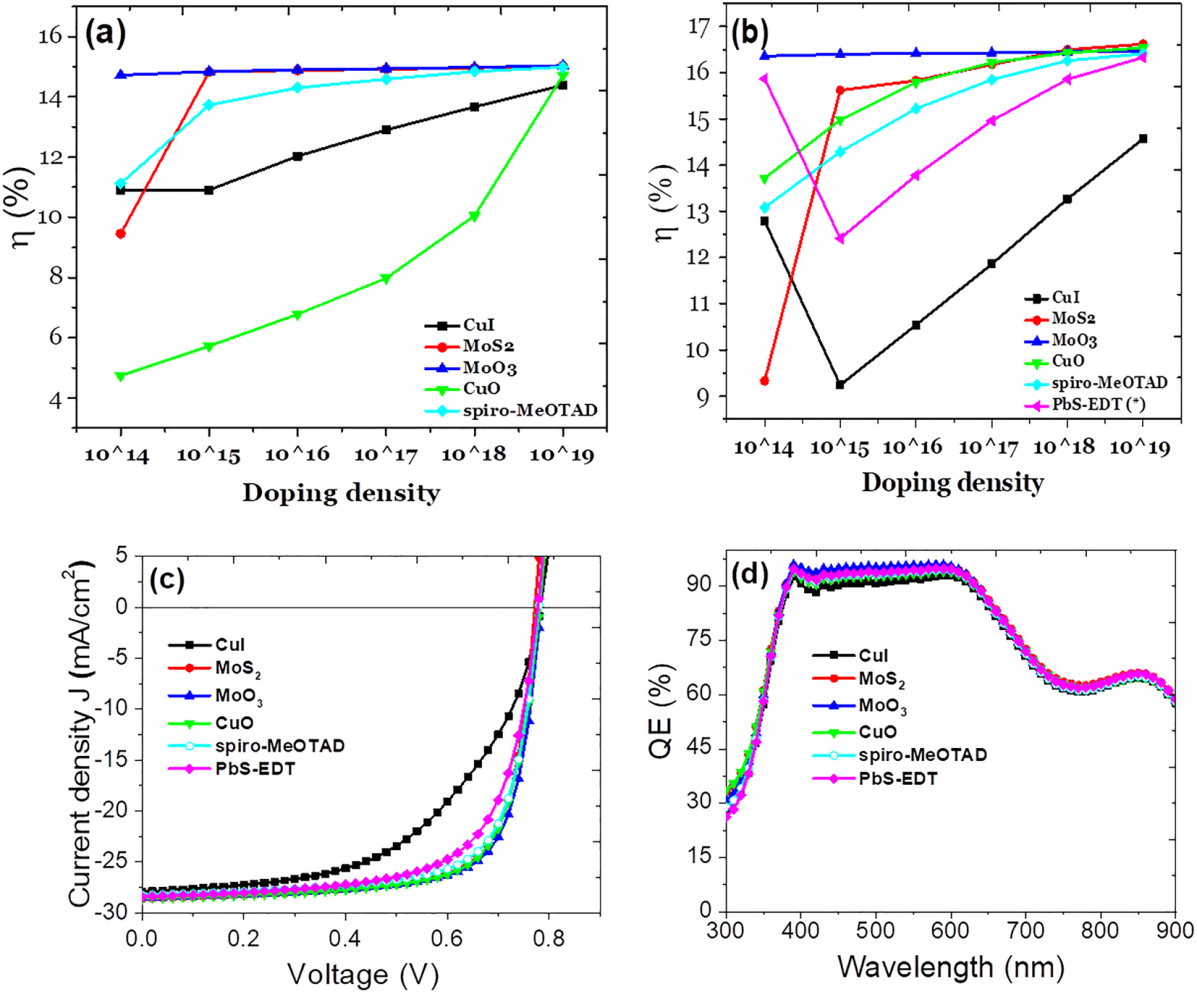
Variation of device efficiency with varying acceptor doping density in (**a**) TiO_2_ (80 nm)/PbS-TBAI (20 nm)/PbS-EDT (45 nm)/HTL/Au (Architecture—1) with different hole transport layers. (**b**) TiO_2_/PbS-TBAI/HTL/Au (Architecture—2) with different HTLs. (**c**) J–V characteristics and (**d**) corresponding QE characteristics of device architecture ITO/TiO_2_(80 nm)/PbS-TBAI (265 nm)/HTL (10 nm)/Au (Architecture—2) with different HTLs at fixed 10^17^ cm^−3^ acceptor doping density.

First we have simulated variation in efficiency of devices with different HTLs by varying their acceptor doping density from 10^14^ to 10^19^ cm^−3^ as shown in Fig. 5a. Among five different HTLs, it has been observed that the conventional device with MoO_3_ as HTL has stable performance with acceptor density variation due to better passivation with the previous PbS-EDT layer. Other HTLs converge towards better device efficiency with increase in acceptor donor density of active layer due to improvement in charge carrier with increase in doping density. Interestingly, at low doping density, the device which has CuO as HTL starts with poor performance and efficiency nearly about 5% due to low J_SC_ and FF resulting into poor device performance. But at large acceptor doping densities such as 10^19^ cm^−3^, the efficiency of this device is found to reach up to the same level as that of devices with other HTLs. This must be due to large concentration of holes at large doping density of the HTL hence the performances of the device improved.

Further, with the simulation we have also tried to investigate performance of devices with different HTLs without PbS-EDT layer before PbS-TBAI layer i.e. the architecture ITO/TiO_2_(80 nm)/PbS-TBAI (265 nm)/HTL (10 nm)/Au (Architecture—2). To keep the effective thickness of the device constant in both architectures, we have taken the thickness of PbS-TBAI layer as 265 nm in the architecture-2. The variation of performances of the devices with increase in doping density of HTL from 10^14^ to 10^19^ cm^−3^ incorporating different HTLs is shown in Fig. 5b. We observed similar trend of increase in V_OC_, Jsc and FF which is resulting in increase in overall efficiency as we move ahead from low acceptor density towards high acceptor doping density. Also the conventional device architecture has been included in graph whose overall efficiency is lower as compared to other devices due to lower J_SC_ and FF even if the device has stable V_OC_ over variation in doping density. Also, there is surprisingly improved performance in CuO based HTL device as compared to previous architecture—1, the efficiency in this architecture is found to be increased from 6 to 15% at 10^15^ cm^−3^ doping density. This improvement is due to improved collection of charge carriers across the layers of the device which is due to the absence of the PbS-EDT layer. The removal of PbS-EDT layers makes the active layer to come in direct engagement with the CuO HTL which is enhancing the current density and hence the device efficiency.

The *J–V* characteristics and corresponding quantum efficiency curve in light conditions of device architecture ITO/TiO_2_(80 nm)/PbS-TBAI (265 nm)/HTL (10 nm)/Au (Architecture—2) with different HTLs such as CuI, MoS_2_, MoO_3_, Spiro-MeOTAD and CuO including PbS-EDT at fixed 10^17^ cm^−3^ acceptor doping density are shown in the Fig. 5c and d respectively. All the devices show very good *J–V* characteristics. The photovoltaic performance parameters of all the devices at constant acceptor doping density under AM 1.5G 1sun spec, 300 K temperature and under identical working condition has shown in the Table 2. It is quite clear that V_OC_ and J_SC_ of the all the devices with different HTLs are almost similar and only variation in FF of the devices with different HTLs. The J_sc_ variation due to different HTLs is further confirmed from the quantum efficiency curve in the Fig. 5d. The device with MoO_3_ HTL device shows highest power conversion efficiency of 16.43% with V_OC_ of 0.783 V and J_sc_ of 28.50 mA cm^−2^ and FF of 73.61%, the corresponding QE spectra also shows similar trend as like J–V curve. If we compare the device performances between ITO/TiO_2_ (80 nm)/PbS-TBAI (220 nm)/PbS-EDT (45 nm)/HTL (10 nm)/Au)—architecture 1 by having different HTLs and ITO/TiO_2_ (80 nm)/PbS-TBAI (220 nm)/HTL/Au—architecture 2, it was found that except CuI as HTL, all other devices with architecture—2 performed better in comparison to the devices with architecture—1. The comparative variation in efficiency of devices of architecture—1 and architecture—2 with IDD also shows that the device having HTLs without PbS-EDT layer (architecture—2) performs better than the architecture with PbS-EDT layer (Architecture—1). Therefore, from this simulation work, it is clear that device architecture 2 which utilizes HTLs without PbS-EDT layer in between the active PbS-TBAI layer and HTL is much better as compared to conventional device architecture—1.

**Table 2:** Performance parameters for device architecture of ITO/TiO_2_(80 nm)/PbS-TBAI (265 nm)/HTL (10 nm)/Au with various HTLs at constant acceptor doping density.

HTL	V_OC_ (V)	J_SC_ (mA cm^−2^)	FF (%)	Efficiency (%)
CuI	0.783	27.91	54.27	11.86
MoS_2_	0.773	28.60	73.19	16.18
MoO_3_	0.783	28.50	73.61	16.43
CuO	0.782	28.58	72.56	16.22
Spiro-MeOTAD	0.780	28.24	71.97	15.84
PbS-EDT	0.778	28.42	67.65	14.96

## Conclusion

In summary, the performance of PbS CQDs based solar cell in different device architectures have been investigated by numerical simulation method. A detailed comparative study has been carried out to analyze the impact of different hole transport layers on device performance. It was observed that the ITO/TiO_2_/PbS-TBAI/HTL/Au device architecture (Architecture—2) in which the PbS-EDT layer has been replaced with different HTLs is superior to the most conventional device architecture of ITO/TiO_2_/PbS-TBAI/PbS-EDT/HTL/Au (Architecture—1). The device with MoO_3_ HTL showed the highest power conversion efficiency of 16.43% with V_OC_ of 0.783 V and Jsc of 28.50 mA cm^−2^ and FF of 73.61%. Besides variation in doping density, the performance of the devices has also been examined with varying defect density at the interface PbS-TBAI/TiO_2_ and a similar trend of decline in performance has been observed. Results carried out in the current study can be used more widely to engineer the device architectures with better HTL by selectively choosing different HTLs and also their variation with doping density and interface defects to uplift the performance of PbS CQD based solar cells.

## Data Availability

The datasets used and/or analysed during the current study available from the corresponding author on reasonable request.
